# Potential of Thermolysin-like Protease A69 in Preparation of Bovine Collagen Peptides with Moisture-Retention Ability and Antioxidative Activity

**DOI:** 10.3390/md19120676

**Published:** 2021-11-27

**Authors:** Jun-Hui Cheng, Xiao-Yu Zhang, Zhen Wang, Xia Zhang, Shi-Cheng Liu, Xiao-Yan Song, Yu-Zhong Zhang, Jun-Mei Ding, Xiu-Lan Chen, Fei Xu

**Affiliations:** 1 State Key Laboratory of Microbial Technology, Shandong University, Qingdao 266237, China; 201820299@mail.sdu.edu.cn (J.-H.C.); 201932456@mail.sdu.edu.cn (X.-Y.Z.); 202012700@mail.sdu.edu.cn (Z.W.); xysong@sdu.edu.cn (X.-Y.S.); 2 College of Marine Life Sciences, and Frontiers Science Center for Deep Ocean Multispheres and Earth System, Ocean University of China, Qingdao 266003, China; zhangyz@sdu.edu.cn; 3 Laboratory for Marine Biology and Biotechnology, Pilot National Laboratory for Marine Science and Technology, Qingdao 266237, China; 4 Department of Molecular Biology, Qingdao Vland Biotech Inc., Qingdao 266102, China; zhangx@vlandgroup.com (X.Z.); liusc@vlandgroup.com (S.-C.L.); 5 Marine Biotechnology Research Center, State Key Laboratory of Microbial Technology, Shandong University, Qingdao 266237, China; 6 Engineering Research Center of Sustainable Development and Utilization of Biomass Energy, Ministry of Education, Yunnan Normal University, Kunming 650500, China

**Keywords:** thermolysin-like protease, bovine bone collagen, collagen peptides, collagen peptide preparation, bioactivity

## Abstract

Bovine bone is rich in collagen and is a good material for collagen peptide preparation. Although thermolysin-like proteases (TLPs) have been applied in different fields, the potential of TLPs in preparing bioactive collagen peptides has rarely been evaluated. Here, we characterized a thermophilic TLP, A69, from a hydrothermal bacterium *Anoxybacillus caldiproteolyticus* 1A02591, and evaluated its potential in preparing bioactive collagen peptides. A69 showed the highest activity at 60 °C and pH 7.0. We optimized the conditions for bovine bone collagen hydrolysis and set up a process with high hydrolysis efficiency (99.4%) to prepare bovine bone collagen peptides, in which bovine bone collagen was hydrolyzed at 60 °C for 2 h with an enzyme–substrate ratio of 25 U/g. The hydrolysate contained 96.5% peptides that have a broad molecular weight distribution below 10000 Da. The hydrolysate showed good moisture-retention ability and a high hydroxyl radical (•OH) scavenging ratio of 73.2%, suggesting that the prepared collagen peptides have good antioxidative activity. Altogether, these results indicate that the thermophilic TLP A69 has promising potential in the preparation of bioactive collagen peptides, which may have potentials in cosmetics, food and pharmaceutical industries. This study lays a foundation for the high-valued utilization of bovine bone collagen.

## 1. Introduction

Collagen peptides have drawn increasing attention due to their various bioactive properties, such as angiotensin I-converting enzyme (ACE-I) inhibitory activity, antioxidant activity, immunomodulatory and antimicrobial activities [1,2,3,4,5]. Studies have shown that collagen peptides display beneficial effects on human health, including improving skin health, muscle strength and bone density [6,7,8], as well as reducing obesity, joint pain and blood pressure [9,10,11]. Due to their various functions, collagen peptides have been applied in cosmetics, food, healthcare and pharmaceutical industries [12,13,14].

Collagen peptides are usually made from collagen-rich animal tissues, including skin, bones, tendons and ligaments. Collagen peptides are generally prepared by acidic, alkali or enzymatic hydrolysis [15,16,17]. Compared to acidic and alkali hydrolysis, enzymatic hydrolysis is gentler and causes less environmental contamination. Nowadays, the common proteases for preparation of collagen peptides are alcalase, pepsin, papain, trypsin and α-chymotrypsin [17,18,19,20,21,22,23]. These enzymes belong to serine protease (alcalase) of the S8 family, serine protease (trypsin and α-chymotrypsin) of the S1 family, cysteine protease (papain) of the C1 family or aspartic protease (pepsin) of the A1 family. However, rare metalloproteinases have been used to prepare collagen peptides.

Thermolysin-like proteases (TLPs) are a group of thermostable zinc metalloproteases secreted by bacteria and fungi, which share high sequence and structure homology and belong to the M4 protease family [24,25]. The prototype of TLPs is thermolysin (EC 3.4.24.27) produced by *Bacillus thermoproteolyticus* [26,27]. The precursor of TLPs usually consists of a signal peptide that is cleaved off during secretion, a propeptide sequence that facilitates folding in enzyme maturation and a catalytic domain [28]. Mature TLPs usually contain only a catalytic domain that has a typical HEXXH motif and a single zinc ion in the active site [29]. TLPs have been applied in various biotechnological and industrial fields. For example, thermolysin and its variants have been widely used in the production of the artificial sweetener aspartame in the food industry [30]. A thermolysin-like neutral protease was added into detergent, which effectively removed proteinaceous stains of textile [31]. In addition, thermolysin has been applied in the diagnosis of animal prion diseases, which can distinguish prion protein PrPC from an abnormal isoform PrPSc [32]. Thus far, there are only a few reports on the potential of TLPs in the preparation of bioactive peptides. Thermolysin has been reported to be used to hydrolyze meat protein [33] and skin collagen [34] from tilapia and skin collagen from goat [35] to produce bioactive peptides with angiotensin-converting enzyme inhibitor activity or antioxidative activity.

*Anoxybacillus caldiproteolyticus* 1A02591 is a protease-secreting thermophilic strain isolated from a deep-sea hydrothermal vent sediment. The most abundant protease secreted by this strain is A69, a TLP sharing 98.8% sequence similarity with thermolysin [36]. In this study, protease A69 was expressed in *Escherichia coli* and the potential of protease A69 in preparing bioactive peptides from bovine bone collagen was evaluated. By characterizing protease A69 and optimizing the hydrolysis conditions of protease A69 on bovine bone collagen, a process to prepare collagen peptides with protease A69 was set up, which showed a high hydrolysis efficiency of more than 99%. The content of peptides in the hydrolysate reached 96.5% and the peptides had a broad molecular weight distribution below 10000 Da. Moreover, the hydrolysate showed good moisture-retention ability and free radical scavenging activity against 1,1-diphenyl-2-picryl-hydrazyl radical (DPPH•), hydroxyl radical (•OH) and superoxide anion (O_2_^−^•), indicating their good bioactivity. The results indicate that TLP A69 has good potential in preparing bioactive peptides from bovine bone collagen.

## 2. Results

### 2.1. Expression, Purification and Characterization of Protease A69

**Figure 1:**
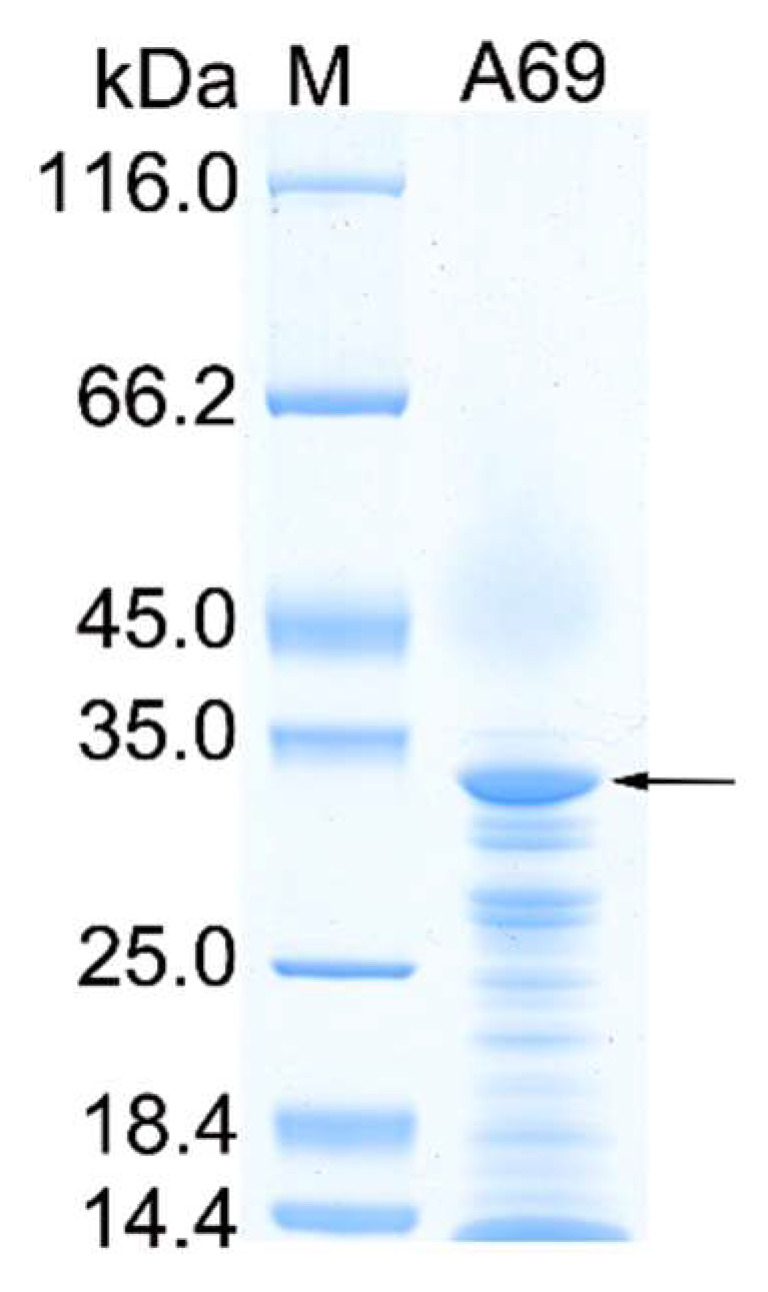
SDS-PAGE analysis of the purified A69. Lane M, protein molecular mass marker; lane A69, the purified recombinant A69. The protein band of A69 is indicated by an arrow.

**Figure 2:**
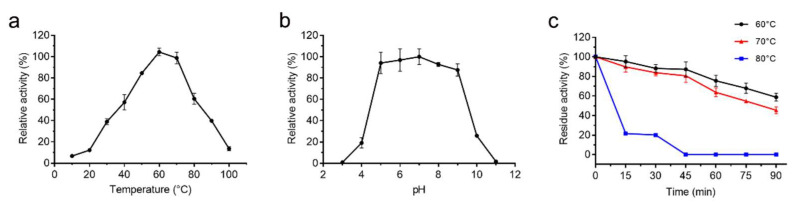
Characterization of protease A69. (**a**) Effect of temperature on the protease activity of A69 toward bovine bone collagen. The activity of A69 was measured in 50 mM Tris-HCl buffer (pH 7.0). The activity of A69 at 60 °C was taken as 100%. (**b**) Effect of pH on the protease activity of A69. The activity of A69 was measured at 60 °C in Britton–Robinson buffers at pH values ranging from 3.0 to 11.0. The activity of A69 at pH 7.0 was taken as 100%. (**c**) Effect of temperature on the protease stability. The residue activity of A69 was measured after the protease was incubated at 60, 70 or 80 °C for different times. The graphs show data from triplicate experiments (mean ± SD).

The gene *A69* amplified from the genome DNA of strain A102591 was cloned into the expression vector pET-22b and the protease A69 was successfully expressed in *E. coli* BL21 (DE3). The recombinant protease A69 was first purified by Ni affinity chromatography and then by gel filtration chromatography. SDS-PAGE analysis showed that the purified protease A69 has an apparent molecular mass of approximately 34 kDa (Figure 1), which is similar to that (34.6 kDa) of thermolysin [37]. To determine the substrate specificity of A69, the activity of purified A69 toward several proteins were measured at 60 °C (Table 1). A69 showed protease activity toward casein, gelatin and bovine bone collagen, but no obvious activity toward elastin. With bovine bone collagen as the substrate, A69 showed the highest activity at 60 °C and retained 13.6% of the highest activity at 100 °C (Figure 2a), indicating that A69 was a thermophilic protease. A69 had protease activity over a broad range from pH 4.0 to 10.0 with a maximal activity at pH 7.0 toward collagen (Figure 2b). Protease A69 retained approximately 60% of its highest activity after incubation at 60 °C for 90 min and 45% after incubation at 70 °C for 90 min (Figure 2c). The half-life time of A69 at 80 °C was approximately 10 min, indicating that A69 has good thermal stability. These results showed that A69 is a thermophilic neutral thermolysin-like protease with collagenolytic activity.

### 2.2. Optimization of the Hydrolysis Parameters of Protease A69 on Bovine Bone Collagen

**Figure 3:**
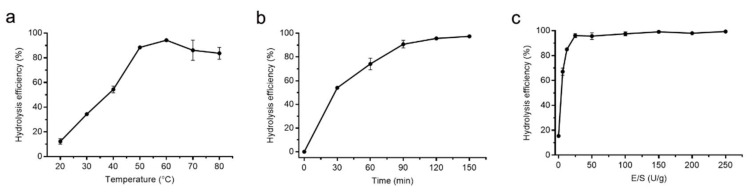
Optimization of the enzymatic hydrolysis parameters of A69 on bovine bone collagen hydrolysis. (**a**) Effect of hydrolysis temperature on enzymatic hydrolysis efficiency. Hydrolysis of A69 on bovine bone collagen was carried out in 50 mM Tris-HCl (pH 7.0) at different temperature (20, 30, 40, 50, 60, 70 or 80 °C). (**b**) Effect of hydrolysis time on enzymatic hydrolysis efficiency. Bovine bone collagen was hydrolyzed with protease A69 for different time (30, 60, 90, 120 or 150 min) at 60 °C. (**c**) Effect of E/S ratio on enzymatic hydrolysis efficiency. Bovine bone collagen was hydrolyzed by protease A69 with different E/S ratio (0, 6.25, 12.5, 25, 50, 100, 150, 200 or 250 U/g) at 60 °C. The graphs show data from triplicate experiments (mean ± SD).

**Figure 4:**
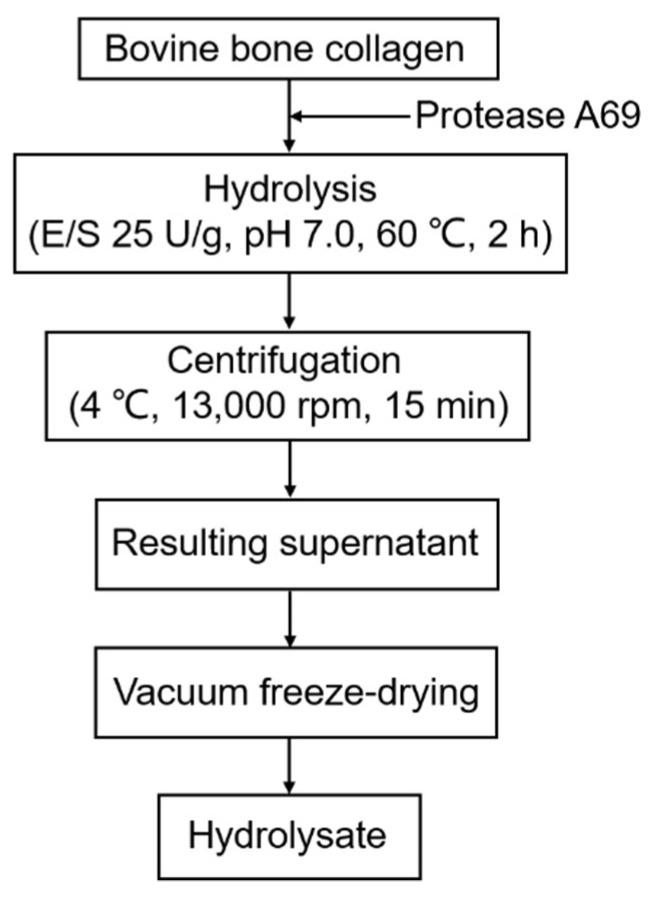
Flow sheet for the preparation of bovine bone hydrolysate with protease A69.

Because protease A69 showed a high activity toward bovine bone collagen (569.95 U/mg), it was very likely to be used to hydrolyze bovine bone collagen for preparation of collagen peptides. Therefore, we tried to prepare peptides from bovine bone collagen with protease A69 as a tool. To determine the optimal conditions for the hydrolysis of A69 on bovine bone collagen, three enzymatic hydrolysis parameters, hydrolysis temperature, hydrolysis time and E/S ratio, were optimized by single factor experiments. As shown in Figure 3, the optimal temperature for the hydrolysis of bovine bone collagen by A69 was 60 °C (Figure 3a), the hydrolysis efficiency increased with hydrolysis time and reached the maximum after 2 h (Figure 3b), and the hydrolysis efficiency reached the maximum when the E/S ratio was ≥25 U/g (Figure 3c). Based on these results, a process for preparation of collagen peptides from bovine bone collagen with protease A69 on the laboratory scale was set up (Figure 4). In this process, the hydrolysis temperature, hydrolysis time and E/S ratio were 60 °C, 2 h and 25 U/g, respectively. With this process, the maximum hydrolysis efficiency of bovine bone collagen reached 99.40%, showing that this process is efficient for bovine bone collagen hydrolysis.

### 2.3. Characterization of Bovine Bone Collagen Hydrolysate

**Figure 5:**
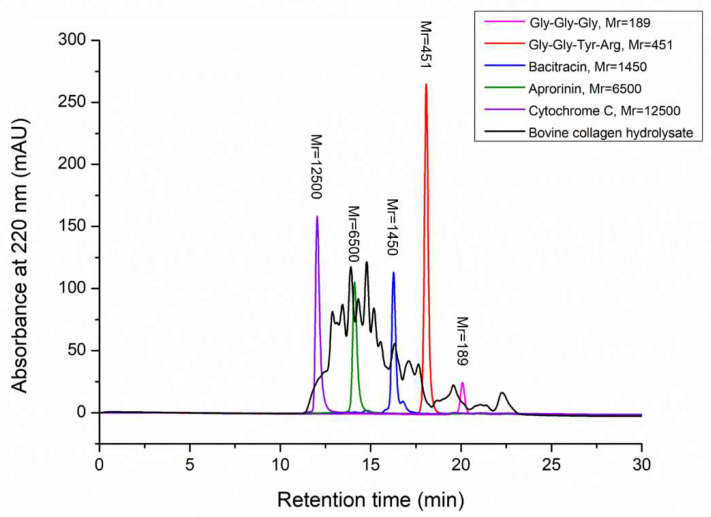
Molecular weight distribution of bovine bone collagen hydrolysate analyzed by HPLC. There are five molecular mass markers: cytochrome C (Mr 12500, purple peak), aprotinin (Mr 6500, green peak), bacitracin (Mr 1450, blue peak), tetrapeptide GGYR (Mr 451, red peak) and tripeptide GGG (Mr 189, pink peak). Molecular weight distribution of bovine bone collagen hydrolysate was shown in black.

To characterize the hydrolysate produced by the hydrolysis of bovine bone collagen with A69, the contents of free amino acids and peptides, amino acid composition and molecular weight distribution of peptides in the hydrolysate were analyzed. Based on the ninhydrin method, there were 3.5% free amino acids and 96.5% peptides in the hydrolysate, showing that the hydrolysate is rich in peptides. The composition of amino acids of the hydrolysate analyzed using automatic amino acid analyzer was summarized in Table 2. The most abundant amino acid among the total amino acids in the hydrolysate was Gly, which accounted for 22.6%. As a unique amino acid in collagen, Hyp accounted for 10.2% in the hydrolysate, indicating that the hydrolysate is rich in collagen peptides. In addition, the contents of Pro, Ala, Arg and Glu were also high. Trp was not detectable due to destruction during hydrochloric acid treatment. Among the free amino acids in the hydrolysate, Cys was the most abundant and accounted for 0.3%, followed by Val, Ile, Leu and Ala, each of which accounted for 0.1%. The molecular weight distribution of peptides in the hydrolysate was analyzed by using HPLC. The result showed that most of the peptides (91.9%) in the hydrolysate have a molecular weight of <10,000 Da, which has a broad molecular weight distribution. Peptides with a molecular weight of <3000 Da accounted for 45.6%, and those with a molecular weight of <1000 Da accounted for 21.1% (Figure 5, Table 3).

### 2.4. Moisture-Absorption and Retention Abilities of Bovine Bone Collagen Hydrolysate

**Figure 6:**
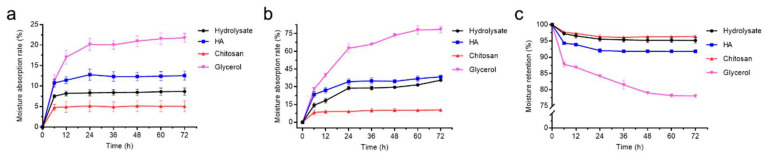
Moisture-absorption and retention abilities of the hydrolysate. (**a**) Moisture-absorption ability in a saturated K_2_CO_3_ container (43% RH) at 25 °C; (**b**) moisture-absorption ability in a saturated (NH_4_)_2_SO_4_ container (81% RH) at 25 °C; (**c**) moisture-retention ability in an allochroic silica gel container at 25 °C. The graphs show data from triplicate experiments (mean ± SD). Differences among groups were considered as significant when *p* < 0.05, which was analyzed by one-way ANOVA test.

To investigate the bioactive properties of the prepared peptides from bovine bone collagen, we measured the R_a_ and R_h_ of the hydrolysate. HA, chitosan and glycerol were used as positive controls because of their good moisture-absorption and retention abilities [38]. The weights of the hydrolysate and the control samples were measured at 43% RH and 81% RH for different times. The R_a_ of the bovine bone collagen hydrolysate at 43% RH was stable after 36 h and the ranking for the R_a_ of all samples was as follows: glycerol > HA > the hydrolysate > chitosan (Figure 6a). The ranking of the R_a_ of the samples at 81% RH was similar to that at 43% RH (Figure 6b). The R_a_ of the hydrolysate was superior to chitosan, reaching 8.7 ± 0.9% at 43% RH and 35.6 ± 1.1% at 81% RH. The R_h_ of the hydrolysate was also stable after 36 h and the ranking for the R_h_ of all samples was as follows: chitosan > the hydrolysate > HA > glycerol (Figure 6c). After 72 h, the R_h_ of the hydrolysate reached 95.2 ± 0.9%, higher than that of HA (91.8 ± 0.3%) and glycerol (78.1 ± 0.5%). These results indicate that the hydrolysate has moderate moisture-absorption ability but good moisture-retention ability.

### 2.5. Antioxidant Activity of Bovine Bone Collagen Hydrolysate

**Figure 7:**
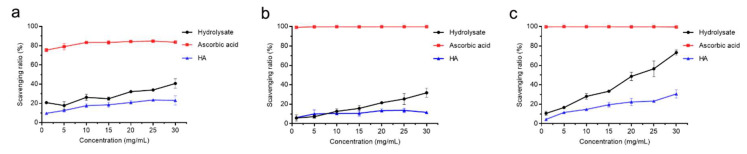
Antioxidant activity of the bovine bone collagen hydrolysate. (**a**) DPPH• scavenging capacity of bovine bone collagen hydrolysate, ascorbic acid and HA. (**b**) O_2_^−^• scavenging capacity of bovine bone collagen hydrolysate, ascorbic acid and HA. (**c**) •OH scavenging capacity of bovine bone collagen hydrolysate, ascorbic acid and HA. The graphs show data from triplicate experiments (mean ± SD). Differences among groups were considered as significant when *p* < 0.05, which was analyzed by one-way ANOVA test.

We further evaluated the antioxidant activity of the hydrolysate by measuring its free-radical scavenging activities towards DPPH•, O_2_^−^• and •OH with HA and ascorbic acid as positive controls. The DPPH• scavenging ratio of the hydrolysate was 40.7% at the concentration of 30 mg/mL, which was lower than that of ascorbic acid (83.6%) but higher than that of HA (23.1%) (Figure 7a). The O_2_^−^• scavenging ratio of the hydrolysate was 31.8% at the concentration of 30 mg/mL, lower than that of ascorbic acid (99.9%) but higher than that of HA (11.6%) (Figure 7b). Especially, the hydrolysate had a significantly higher •OH scavenging ratio (73.2%, *p* < 0.05) than that of HA (30.7%) (Figure 7c). These results showed that the prepared hydrolysate had the capacity to scavenge free radicals and exhibited good antioxidant activity.

## 3. Discussion

Large quantities of byproducts, such as bovine bone, skin and tendons, are produced in the beef processing industry. These byproducts contain a large amount of collagen. According to the report of the China National Beef Cattle Industrial Technology System (China NBCITS) in 2019, the global yield of collagen-rich bovine bone, skin and tendons reached 31.4 million tons [39]. However, most of these byproducts are underutilized. Making full use of these collagen-rich wastes is not only economical, but also conducive to environmental protection. Because bovine bone, skin and tendons are rich in collagen, they are good materials for collagen peptides preparation [40,41,42]. Especially, more than 12 million tons of bovine bone are produced annually in China [43], which is a suitable source for collagen peptides because of its high yields, low cost and high collagen content.

Although TLPs have been shown to have various biotechnological and industrial applications, there are only a few reports on the potential of TLPs in preparing bioactive peptides. Daud et al. (2015) reported that hydrolysates derived from red tilapia meat protein by thermolysin had antioxidative and antihypertensive activities, which were higher than those hydrolyzed by alcalase [33]. Recently, Pa’ee et al. (2021) produced hydrolysate from tilapia skin collagen type I using thermoase PC10F and predicted the potential ACE-inhibitory peptides by in silico analysis [34]; Pratiwi et al. (2021) prepared hydrolysate with MW of 117.5–14.6 kDa from *Kacang* goat skin collagen with thermolysin at 37 °C for 1 h, and found that the <3 kDa fractionation had the angiotensin-converting enzyme inhibitor activity in the range of 36.2–91.3%, with an IC_50_ of 82.94 μg/mL [35]. These studies suggest that TLPs likely have good potentials in preparing bioactive peptides, which, however, needs more study and evaluation.

In this study, we characterized a thermophilic TLP, A69, from *A. caldiproteolyticus* 1A02591, and evaluated its potential in preparing collagen peptides from bovine bone collagen. After optimizing the hydrolysis parameters, we successfully set up a preparation process of bovine bone collagen peptides and the optimal hydrolysis conditions are hydrolysis at 60 °C for 2 h with an E/S ratio of 25 U/g. With this process, the maximum hydrolysis efficiency reached 99.4%, indicating that it was an efficient process for bovine bone collagen hydrolysis. The hydrolysis process was more efficient than bovine collagen hydrolysate prepared from a dual enzyme mixture—the Alcalase/Flavourzyme combination, the degree of hydrolysis (DH) of which after 24 h hydrolysis was 20.4% [44]. It is worth noting that the hydrolysis temperature 60 °C is high enough to prevent most bacterial contamination during the process of collagen hydrolysis. Analysis of the prepared bovine bone collagen hydrolysate showed that it contains 96.5% peptides and 10.2% Hyp, indicating that the hydrolysate is rich in collagen peptides.

Collagen hydrolysates have been effectively utilized due to their good moisturizing properties at the stratum corneum layer of the skin, which reduce the effects of skin dryness, laxity and wrinkles [45]. Eckert et al. (2021) reported that collagen hydrolysates of fish, jellyfish and bovine origin that were used as food supplements could maintain cartilage health of dogs and could be as potential therapeutic drugs in early osteoarthritis [46]. Schadow et al. (2017) used several collagen hydrolysates as nutraceuticals for human osteoarthritis, and found that there were marked differences between collagen hydrolysates of different origins [47]. To investigate the bioactive properties of the prepared bovine bone collagen peptides, we measured the moisture absorption, retention abilities and antioxidant activity of the hydrolysate. Reactive oxygen species (DPPH•, •OH and O_2_^−^•) are highly related to human health, which may cause aging, cancer, inflammation and other diseases [48]. Free radical scavenging is a primary mechanism by which antioxidants inhibit oxidative processes [49]. HA has a good free radical-scavenging ability and has been widely used in the cosmetics industry [50]. We found that the scavenging ratios for DPPH•, •OH and O_2_^−^• of the bovine bone collagen peptides we prepared were much higher than those of HA. Especially, its •OH scavenging ability ratio reached 73.2% at 30 mg/mL, two-fold higher than that of HA (30.7%). The bovine bone collagen peptides we prepared have a higher DPPH• scavenging rate (40.7%) than that of bovine bone collagen peptides prepared by recombinant collagenase from *Bacillus cereus* (18.9%) and that (31.1%) of a reported shrimp hydrolysate [39,51]. They also have higher •OH scavenging rate (73.2%) than the reported shrimp hydrolysate (26.6%) [51]. In addition, the prepared collagen peptides showed good moisture-retention ability at 25 °C, which may demonstrate this ability when applied on human skin because collagen hydrolysates have been shown to have good moisturizing properties at the stratum corneum layer of skin [45]. Therefore, the bovine bone collagen peptides we prepared likely have potential in cosmetics as an anti-aging and moisturizing ingredient. Due to its good antioxidative activity, it may also be used as functional food ingredient, healthcare products and pharmaceuticals to improve health of human and animals and prevent disease.

Collagen hydrolysates are usually heterogeneous mixtures of collagen fragments. HPLC is a common method to determine the molecular weight range of peptides in collagen hydrolysates [52]. Atomic force microscopy (AFM) and diffusion-ordered NMR spectroscopy have also been used to determine the molecular organization of collagen fragments [53]. It has been shown that collagen peptides with different molecular weights may have different bioactivities and functional properties and be applied in various industries. Low-molecular-weight peptides (molecular weight of <1000 Da) are preferred in cosmetics, functional food and nutraceuticals because they are absorbed efficiently by the human body [54]. High-molecular-weight peptides contribute to the stability of emulsion, and are more efficient in reducing the interfacial tension due to unfolding and reorientation at the interface [55,56,57]. Our result showed that the peptides prepared from bovine bone collagen with A69 had a broad molecular weight distribution, in which peptides with a molecular weight of <1000 Da accounted for 21.1%, those with a molecular weight of 1000–3000 Da accounted for 24.5%, and those with a molecular weight of 3000–10,000 Da accounted for 46.3%. In the future, the peptides with different molecular weight ranges in the hydrolysate may be separated, and products of collagen peptides with different molecular weight ranges were prepared, which may be applied in different industries according to their different bioactivities and functional properties.

## 4. Materials and Methods

### 4.1. Experimental Materials

Bovine bone collagen was purchased from Kinry Biotech Co.,Ltd. (Jinan, China). Casein, gelatin, elastin, aprotinin, cytochrome C, salicylic acid and pyrogallol were purchased from Sigma (St. Louis, MO, USA). Bacitracin and chitosan were purchased from Aladdin (Shanghai, China). Tetrapeptide GGYR and tripeptide GGG were synthesized by Qiangyao Co., Ltd. (Shanghai, China). Ascorbic acid and glycerol were purchased from Sinopharm Chemical Reagent Co., Ltd. (Shanghai, China). DPPH• was purchased from Tokyo Chemical Industry (Tokyo, Japan). Other chemicals were of analytical grade and commercially available.

### 4.2. Expression and Purification of Protease A69

The gene sequence of protease A69 (WP_181554874.1) was cloned from the genomic DNA of *Anoxybacillus caldiproteolyticus* A102591 (MCCC1A02591) by PCR and inserted into the *Nde*I and *Xho*I sites of pET-22b (+) to construct the expression vector pET-22b-*A69*. Then pET-22b-*A69* was transformed into *E. coli* BL21(DE3), and the transformant was cultured at 37 °C and 180 rpm in Luria-Bertani (LB) liquid medium containing 100 mg/mL ampicillin. When the OD_600_ of cells in the culture reached approximately 0.6, 0.5 mM isopropyl-D-thiogalactopyranoside (IPTG) was added in the culture as an inducer, and the culture was further incubated at 18 °C and 110 rpm for 14 h. Then, the cells in the culture were harvested by centrifugation (6000 rpm, 10 min), resuspended in lysis buffer (50 mM Tris-HCl, 100 mM NaCl, pH 8.0) and disrupted by pressure three times. The recombinant protease A69 was purified by Ni affinity chromatography and then by gel filtration chromatography on a Superdex G200 column (GE, Boston, MA, USA). The purified protease A69 was analyzed by 12.5% sodium dodecyl sulfate–polyacrylamide gel electrophoresis (SDS-PAGE). Protein concentration of A69 was determined by using the Pierce BCA protein assay kit (Thermo Scientific, Waltham, MA, USA) and calibrated with bovine serum albumin at different concentrations.

### 4.3. Enzyme Assays

The activity of A69 toward casein was assayed at 60 °C using the Folin-phenol method [58]. The reaction mixture contained 100 μL enzyme solution and 100 μL of 2% (*w*/*v*) casein. After incubation at 60 °C for 10 min, the reaction was terminated by an addition of 200 μL trichloroacetic acid (0.4 M). After centrifugation at 13,000 rpm for 10 min, 100 μL of the supernatant was reacted with 500 μL of sodium carbonate solution (0.4 M) and 100 μL of the Folin–phenol reagent at 40 °C for 20 min, and then the OD_660_ of the mixture was measured. One unit of enzyme activity (U) was defined as the amount of enzyme that released 1 μg tyrosine from casein per min. The activities of A69 toward collagen and gelatin were measured by a modified method as previously described [59]. For collagen, 1 mL enzyme solution was incubated with 10 mg bovine bone collagen for 1 h at 60 °C with continuous stirring. One unit of enzyme activity (U) was defined as the amount of enzyme that released 1 μmol leucine from collagen per hour. For gelatin, 100 μL enzyme solution was incubated with 100 μL of 2% (*w*/*v*) gelatin at 60 °C for 10 min. One unit of enzyme activity (U) was defined as the amount of enzyme that released 1 μmol of leucine from gelatin per hour. The elastinolytic activity of protease A69 was determined with the method as previously described [60]. The enzyme solution was incubated with 5 mg elastin–orcein at 60 °C for 1 h, and then the residual elastin–orcein was removed by centrifugation. The OD_590_ of the supernatant was recorded. One unit of enzyme activity (U) was defined as the amount of enzyme that released 1 nmol orcein per min.

### 4.4. Characterization of Protease A69

Substrate specificity of protease A69 was determined by measuring its activities toward casein, bovine bone collagen, gelatin and elastin. To determine the effect of temperature on the protease activity, the protease activity of A69 was measured in 50 mM Tris-HCl buffer (pH 7.0) from 0 to 100 °C with bovine bone collagen as substrate. To determine the optimal pH of protease A69, the activity of A69 was determined at 60 °C in Britton–Robinson buffer at pH values ranging from 3.0 to 11.0. To evaluate the thermal stability of protease A69, the residual activity was measured at 60 °C and pH 7.0 after A69 was incubated at 60, 70 or 80 °C for different time intervals (15, 30, 45, 60, 75 or 90 min).

### 4.5. Optimization of Enzymatic Hydrolysis Conditions of A69 on Bovine Bone Collagen

To determine the optimal hydrolysis conditions of protease A69 on bovine bone collagen, three parameters, hydrolysis temperature, hydrolysis time and enzyme–substrate ratio (E/S), were optimized by single-factor experiments. To determine the optimal hydrolysis temperature, hydrolysis of A69 on bovine bone collagen was carried out in 50 mM Tris-HCl (pH 7.0) at different temperature (20, 30, 40, 50, 60, 70 or 80 °C) with constant agitation (180 rpm) for 2 h with an E/S ratio of 25 U/g. The optimal hydrolysis time was determined by hydrolyzing bovine bone collagen with protease A69 for different times (30, 60, 90, 120 or 150 min) at 60 °C with an E/S ratio of 25 U/g. To determine the optimal E/S ratio, 10 mg bovine bone collagen was reacted with 1 mL enzyme solution with different E/S ratio (0, 6.25, 12.5, 25, 50, 100, 150, 200 or 250 U/g) at 60 °C for 2 h. Hydrolysis reaction was terminated by heating the reaction mixture at 100 °C for 15 min. The reaction mixture was centrifuged (13,000 rpm, 4 °C, 15 min) and the residual collagen was freeze dried and weighed. The supernatant was the collagen hydrolysate, which was collected and freeze dried for further analysis. The hydrolysis efficiency was calculated using the equation as follows:

Hydrolysis efficiency (%) = (W_a_ − W_b_)/W_a_ × 100 (1)

where W_a_ and W_b_ are the weight of the samples before and after being hydrolyzed.

### 4.6. Analysis of Composition of Amino Acids and Content of Peptides in Bovine Bone Collagen Hydrolysate

Five mg freeze-dried bovine bone collagen hydrolysate was dissolved in 1 mL ddH_2_O and trifluoroacetic acid (*v*/*v*, 1%) was added to precipitate potential proteins. The solution was incubated at 25 °C for 30 min, and then was centrifuged (13,000 rpm, 4 °C, 10 min) to remove the precipitated proteins. The supernatant was collected to analyze the contents of free amino acids and peptides and the composition of amino acids in the bovine bone collagen hydrolysate. Before total amino acids of the hydrolysate was analyzed, the sample was hydrolyzed with 6.0 M HCl at 110 °C for 22 h [61], and HCl was volatilized with a rotary evaporator. Then the sample was redissolved in ddH_2_O. Before free amino acids of the hydrolysate were analyzed, sulfosalicylic acid (4%, *w*/*v*) was added to the sample and the sample was incubated at 25 °C for 30 min. Then the sample was centrifuged (13,000 rpm, 4 °C, 10 min) to remove the precipitated peptides. The contents of amino acids in the treated samples were determined by ninhydrin method [62]. The composition of amino acids in the treated samples was analyzed using automatic amino acid analyzer HITACHI 835 (Hitachi, Ltd., Tokyo, Japan). The content of peptides in the hydrolysate was determined by subtracting the content of free amino acids from that of total amino acids.

### 4.7. Analysis of Molecular Weight Distribution of Bovine Bone Collagen Hydrolysate

The molecular weight distribution of collagen hydrolysate was analyzed by high-performance liquid chromatography (HPLC, Shimadzu, Kyoto, Japan) equipped with a TSK gel G2000 SW_XL_ column (7.8 × 300 mm, Tosoh, Tokyo, Japan) according to the method previously described [63]. The mobile phase used was 45% acetonitrile containing 0.1% (*v*/*v*) trifuoroacetic acid. HPLC was performed at a flow rate of 0.5 mL/min and monitored at 220 nm at 30 °C. The molecular weight distribution was calibrated with five molecular mass markers: cytochrome C (Mr 12500), aprotinin (Mr 6500), bacitracin (Mr 1450), tetrapeptide GGYR (Mr 451) and tripeptide GGG (Mr 189). The area of sample chromatograph was integrated at different ranges (<1000 Da, 1000–3000 Da, 3000–5000 Da, 5000–10,000 Da and >10,000 Da). The proportion of each range of peptides in the hydrolysate were expressed as the percentage of area of corresponding molecular weight range to the total chromatograph area.

### 4.8. Analysis of the Moisture-Absorption and Retention Abilities of Bovine Bone Collagen Hydrolysate

The moisture-absorption and retention abilities of the hydrolysate were measured by the method previously described [19]. Hyaluronic acid (HA), chitosan and glycerol were used as the control samples. Before the moisture-absorption test, the hydrolysate and the control samples were dried for 24 h. Then, 50 mg dried samples were put in an airtight container (43% relative humidity, RH) with saturated K_2_CO_3_ and an airtight container (81% RH) with saturated (NH_4_)_2_SO_4_ at 25 °C. The samples were weighed after 6, 12, 24, 36, 48, 60 and 72 h. The water-absorption ability (R_a_) was calculated by using the following equation:

R_a_ (%) = (W_n_ – W_0_)/W_n_ × 100 (2)

where W_0_ and W_n_ are the weight of the samples before and after being put in the airtight container for an indicated time.

For measuring the moisture retention abilities of the hydrolysate, the hydrolysate and the control samples in 43% RH chamber were put in another airtight container with allochroic silica gel at 25 °C. The samples were weighed after 6, 12, 24, 36, 48, 60 and 72 h. The water-retention ability (R_h_) was calculated by using the following equation:

R_h_ (%) = (H_n_/H_0_) × 100 (3)

where H_0_ and H_n_ are the weight of the samples before and after being put in the airtight container with allochroic silica gel for an indicated time.

### 4.9. Analysis of the Antioxidant Activity of Bovine Bone Collagen Hydrolysate

The antioxidant activity of the bovine bone collagen hydrolysate was determined by measuring its activity to scavenge DPPH•, •OH and O_2_^−^•. HA and ascorbic acid, which can scavenge radicals, were used as positive controls. In the assay, 100 μL hydrolysate at different concentrations (1, 5, 10, 15, 20, 25 and 30 mg/mL) was mixed with 200 μL of 0.1 mM DPPH in 50% ethanol. To determine the background absorbance, DPPH solution was replaced with an equal volume of 50% ethanol solution. In blank, the sample was replaced with an equal volume of H_2_O. The mixture was incubated in the dark for 40 min at 25 °C, and then the absorbance of the mixture at 525 nm was recorded.

The •OH scavenging activity of the hydrolysate was measured by a modified method as previously reported [64]. HA and ascorbic acid were used as positive controls. In the assay, 200 μL hydrolysate at different concentrations (1, 5, 10, 15, 20, 25 and 30 mg/mL) was mixed with 200 μL FeSO_4_ solution (9 mM) and 200 μL ethanol solution of salicylic acid (9 mM). Then, 200 μL H_2_O_2_ solution (8.8 mM) was added to the mixture to start the reaction. To determine the background absorbance, H_2_O_2_ solution was replaced with an equal volume of H_2_O. In blank, the sample was replaced with an equal volume of H_2_O. After incubation at 37 °C for 30 min, the absorbance of the mixture at 510 nm was determined.

The O_2_^−^• scavenging activity of the hydrolysate was measured by the pyrogallol autoxidation method [65]. HA and ascorbic acid were used as positive controls. Briefly, 200 μL hydrolysate at different concentrations (1, 5, 10, 15, 20, 25 and 30 mg/mL) was mixed with 80 μL HCl solution (10 mM) of pyrogallol (25 mM) and 900 μL Tris-HCl buffer (50 mM, pH 8.2). To determine the background absorbance, pyrogallol solution was replaced with an equal volume of HCl solution (10 mM). In blank, the sample was replaced with an equal volume of H_2_O. After incubation at 25 °C for 5 min, 200 μL HCl solution (8 mM) was added to terminate the reaction, and then the absorbance of the mixture at 320 nm was determined. All experiments were carried out in triplicate. The free radical-scavenging activity (D) was calculated by using the following equation:

D (%) = [1– (A_i_ − A_j_)/A_0_] × 100 (4)

where A_i_ is the absorbance of the sample, A_j_ is the background absorbance and A_0_ is the absorbance of the blank control.

### 4.10. Statistics Analysis

Data are presented as the arithmetic mean ± SD of triplicate samples. One-way analysis of variance (ANOVA) was applied for all the experiments, where GraphPad Prism 7 was used for statistical calculations (Graph pad Software, San Diego, CA, USA). *p* < 0.05 was considered statistically significant.

## 5. Conclusions

In this study, the potential of a thermophilic TLP A69 in preparation of bovine collagen peptides was evaluated. A69 showed the highest activity towards bovine collagen at 60 °C and pH 7.0. By optimizing the hydrolysis conditions of protease A69 on bovine bone collagen, a process to prepare collagen peptides with protease A69 was set up, which showed a high hydrolysis efficiency of more than 99%. The hydrolysate had a high content of peptides of 96.5% and the peptides had a broad molecular weight distribution below 10,000 Da. Moreover, the bovine collagen peptides showed good moisture-retention ability and free radical scavenging activity against DPPH•, •OH and O_2_^−^•. The results indicate that TLP A69 has a good potential in preparing bioactive peptides from bovine bone collagen, and the prepared peptides may have potentials in cosmetics, food and pharmaceutical industries.

## Figures and Tables

**Table 1:** The substrate specificity of protease A69.

Substrate	Specific Activity *^a^* (U/mg)
Casein	327.32 ± 9.24
Bovine bone collagen	569.95 ± 29.27
Gelatin	686.52 ± 18.26
Elastin–orcein	Not detectable

*^a^* The specific activity of A69 toward each substrate was measured at 60 °C and pH 7.0. The data shown in the table are from triplicate experiments (mean ± SD).

**Table 2:** Composition and content of amino acids in the bovine collagen hydrolysate.

Amino Acid	Free Amino Acids (g/100 g)	Total Amino Acids (g/100 g)
Asp	0.008 ± 0.001	5.318 ± 0.227
Thr	0.017 ± 0.001	2.310 ± 0.065
Ser	0.024 ± 0.001	3.364 ± 0.147
Glu	0.009 ± 0.003	6.957 ± 0.241
Gly	0.048 ± 0.002	22.558 ± 0.845
Ala	0.114 ± 0.001	8.625 ± 0.367
Cys	0.273 ± 0.012	0.406 ± 0.002
Val	0.144 ± 0.002	1.934 ± 0.065
Met	0.056 ± 0.001	0.361 ± 0.030
Ile	0.142 ± 0.002	1.327 ± 0.042
Leu	0.141 ± 0.003	2.971 ± 0.124
Tyr	-	0.582 ± 0.046
Phe	-	1.959 ± 0.091
Lys	0.017 ± 0.002	3.520 ± 0.157
His	0.006 ± 0.001	0.558 ± 0.023
Trp *^a^*	-	-
Arg	0.024 ± 0.002	7.428 ± 0.355
Pro	-	12.792 ± 0.542
Hyp	-	10.228 ± 0.442
Hyl	-	0.056 ± 0.079
Total	1.022 ± 0.019	93.254 ± 3.889

*^a^* Trp was not detectable because it was destroyed in the process of acid hydrolysis. The data shown in the table are from triplicate experiments (mean ± SD).

**Table 3:** Peptide molecular weight distribution of bovine collagen peptides hydrolyzed by protease A69.

MW Range (Da)	Content *^a^* (%)
>10,000	8.07
5000–10,000	26.05
3000–5000	20.27
1000–3000	24.53
<1000	21.08

*^a.^* The content of each range of peptides in the bovine collagen hydrolysate were calculated based on the percentage of the area of corresponding molecular weight range in the total chromatograph area of the hydrolysate in the HPLC chromatogram.

## Data Availability

The amino acid sequence of protease A69 has been submitted to NCBI database under the accession number WP_181554874.1. It can be found here: https://www.ncbi.nlm.nih.gov/protein/WP_181554874.1/.
